# Intra-operator Repeatability of Manual Segmentations of the Hip Muscles on Clinical Magnetic Resonance Images

**DOI:** 10.1007/s10278-022-00700-0

**Published:** 2022-10-11

**Authors:** Giorgio Davico, Francesca Bottin, Alberto Di Martino, Vanita Castafaro, Fabio Baruffaldi, Cesare Faldini, Marco Viceconti

**Affiliations:** 1 Department of Industrial Engineering (DIN), Alma Mater Studiorum – University of Bologna, Bologna, Italy; 2 Laboratorio di Tecnologia Medica, IRCCS Istituto Ortopedico Rizzoli, Bologna, Italy; 3 Clinica Ortopedica e Traumatologica I, IRCCS Istituto Ortopedico Rizzoli, Bologna, Italy; 4 Department of Biomedical and Neuromotor Sciences (DIBINEM), Alma Mater Studiorum – University of Bologna, Bologna, Italy

**Keywords:** Manual segmentation, MRI, Muscles, Pathological population, Repeatability

## Abstract

The manual segmentation of muscles on magnetic resonance images is the gold standard procedure to reconstruct muscle volumes from medical imaging data and extract critical information for clinical and research purposes. (Semi)automatic methods have been proposed to expedite the otherwise lengthy process. These, however, rely on manual segmentations. Nonetheless, the repeatability of manual muscle volume segmentations performed on clinical MRI data has not been thoroughly assessed. When conducted, volumetric assessments often disregard the hip muscles. Therefore, one trained operator performed repeated manual segmentations (*n* = 3) of the iliopsoas (*n* = 34) and gluteus medius (*n* = 40) muscles on coronal T1-weighted MRI scans, acquired on 1.5 T scanners on a clinical population of patients elected for hip replacement surgery. Reconstructed muscle volumes were divided in sub-volumes and compared in terms of volume variance (normalized variance of volumes – nVV), shape (Jaccard Index—JI) and surface similarity (maximal Hausdorff distance—HD), to quantify intra-operator repeatability. One-way repeated measures ANOVA (or equivalent) tests with Bonferroni corrections for multiple comparisons were conducted to assess statistical significance. For both muscles, repeated manual segmentations were highly similar to one another (nVV: 2–6%, JI > 0.78, HD < 15 mm). However, shape and surface similarity were significantly lower when muscle extremities were included in the segmentations (e.g., iliopsoas: HD –12.06 to 14.42 mm, *P* < 0.05). Our findings show that the manual segmentation of hip muscle volumes on clinical MRI scans provides repeatable results over time. Nonetheless, extreme care should be taken in the segmentation of muscle extremities.

**Supplementary Information:**

The online version contains supplementary material available at 10.1007/s10278-022-00700-0.

## Introduction

The quantification of skeletal muscle volume using MRI is used in a number of clinical and research applications such as sport medicine [1], the quantification of sarcopenia [2], or the generation of patient-specific musculoskeletal dynamics models [3, 4]. While in the clinical routine, the simple quantification of a single muscle cross-sectional area may be sufficient to evaluate the loss of muscle tissue [5, 6], research applications usually require that the entire muscle volume is segmented in the MRI images. This operation is cumbersome and time-consuming, which is why there is intense research on the automation of this operation [7–11]. However, all automatic segmentation algorithms are validated assuming the manual segmentation as the true value [12, 13]; thus, it becomes very important to quantify the repeatability of the manual segmentation of skeletal muscle volume on MRI images.

A substantial amount of work has already been done on the quantification of the reliability and repeatability of skeletal muscle volumes manually segmented on MRI images. An excellent systematic review of this literature is reported here [14]. Overall, the manual segmentation of skeletal muscles, performed slice-by-slice, showed good to excellent intra-rater reliability, moderate to good inter-rater reliability and good test–retest reliability. For the above reasons, this technique is currently considered the gold-standard for skeletal muscle segmentation. However, Pons and colleagues highlighted that hip and trunk muscles (e.g. gluteus medius and iliopsoas) were often neglected, which is surprising given the key role these muscles play in the stabilization of the spine and in many activities of daily living [15–18], and only healthy muscles were typically analyzed. Indeed, when assessed, the reliability of manual segmentations for pathological muscles was lower than for healthy muscles [19–21]. Moreover, while slice-by-slice segmentations are commonly used to demonstrate the concurrent validity of novel semi-automatic techniques, only one study on the rotator cuff muscles was conducted to assess the validity of manual segmentations [22], and test–retest repeatability was quantified for the quadriceps and for the upper limb muscles only.

In addition, the repeatability of manual segmentations may highly depend on the specific MRI sequence used to generate the images [23, 24]. Despite new imaging sequences, such as Dixon scans, have been developed to highlight specific features (e.g. fat infiltration) in soft-tissues and muscles, T1-weighted MRI images are typically preferred to assess muscle size and morphology, and fat infiltration [14, 25]. Indeed, T1-weighted images are characterized by excellent anatomical detail and high signal-to-noise ratio (compared to other MRI sequences), which makes them ideal to assess muscles [26, 27]. Another important factor is the field intensity of the MRI system; 3 T systems are becoming widely available, although in most clinical settings 1.5 T systems are still in use. Finally, the region of interest and the location (superficial or deep) of the muscles to be segmented are likely to affect the accuracy of manual muscle segmentations. Thus, it is necessary to conduct a repeatability analysis for the specific region (hip and trunk muscles) and for the specific MRI system and sequence adopted.

To the purpose, one trained operator performed repeated manual segmentations of the iliopsoas and gluteus medius muscle volumes on 1.5 T clinical MRI scans. The twofold aim of the study was (1) to determine if the manual segmentation of hip muscle volumes provides similar results over time, and (2) to understand how the (segmentation) error is distributed across the muscle volume of interest (towards the extremities or in the belly region). More specifically, three hypotheses were tested: (H1) that repeated manual segmentations of the gluteus medius and iliopsoas muscles on MRI show a high level of agreement, (H2) that manual segmentations of the iliopsoas muscle, given its complex geometry [28], are less repeatable than those of the gluteus medius muscle, and (H3) that limiting the segmentations to the muscle belly (quicker to perform compared to full segmentations and typically included in clinical hip joint scans) would be less prone to inaccuracies, as muscle extremities may be difficult to identify on MRIs.

## Materials and Methods

### Data Collection

Medical imaging data were retrospectively collected from the institutional 2015–2020 database. The study was approved by the Institutional Review Board and was conducted in compliance with the Health Insurance Portability and Accountability Act and the Declaration of Helsinki. The temporal threshold was selected to minimize image quality variability due to technological improvements (in MRI acquisition). The dataset was further screened to exclude those MRIs where the iliopsoas and/or the gluteus medius muscles were not visible in their entirety. Thus, medical imaging data on 40 gluteus medius and 34 iliopsoas muscles were included in the study. All selected MRIs were acquired on 1.5 T scanners, with a coronal T1-weighted sequence, but different spatial resolution (512 × 512 pixels, pixel size: 0.817 ± 0.053 mm, min = 0.723 mm, max = 0.938 mm) and slice thickness (min = 4 mm, max = 6 mm) depending on the MRI scanner and year of acquisition (see Table S1 in Supplementary material for more details). These were representative of a heterogeneous pathological population (age: 57.3 ± 19.7 years, mass: 68.9 ± 10.7 kg, male/female ratio: 8/12) of patients candidate for hip replacement surgery on one or both sides of the body (Table 1).

**Table 1:** Demographics of patients and image acquisition details

Patients demographics		Image acquisition	
Population size	20	Magnetic field strength (T)	1.5
Age (years)	57.3 ± 19.7	Sequence	Coronal T1-w
Mass (kg)	71.35 ± 12.45	Echo time (ms)	11.08 ± 1.62
Sex (male/female)	8/12	Repetition time (ms)	553.58 ± 146.47
		Spatial resolution	512 × 512 pixels
Diagnosis	Primary arthrosis (12) Secondary arthrosis (1) Osteonecrosis (5) Femoral fracture (2)	Pixel size (mm)	0.817 ± 0.053 0.723 (min) 0.938 (max)
		Field of view (mm)	418.5 ± 26.50 370.02 (min) 480 (max)
		Slice thickness (mm)	4 (min) 6 (max)

Age, mass and spatial resolution values are reported as mean ± standard deviation

*kg* kilograms, *mm* millimeters, *T* tesla

### Data Processing

**Fig. 1:**
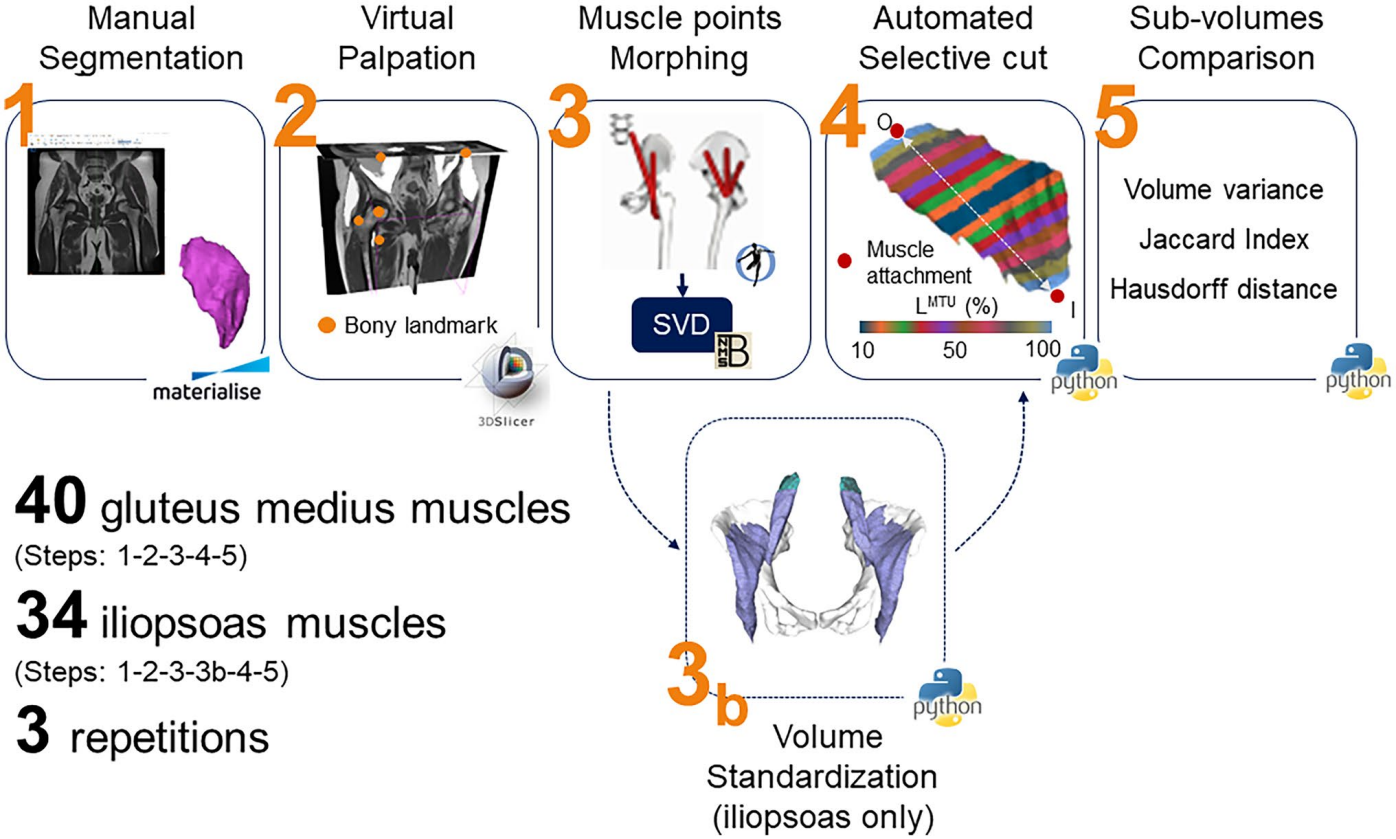
Five-step workflow to process medical imaging data. The workflow comprised of manual segmentation, identification of bony landmarks via virtual palpation, muscle point morphing using single value decomposition (SVD) algorithm, selective cut of the muscle volume, sub-volume comparison computing volume variance, Jaccard index and Hausdorff distance. For the iliopsoas muscle, a further step was performed to standardize segmented muscle volumes prior to cutting. MTU = muscle–tendon unit, I = muscle insertion, O = muscle origin

A five-step workflow was implemented to process all medical imaging data (Fig. 1). This included (1) slice-by-slice manual segmentation of iliopsoas and gluteus medius muscles on MRIs, (2) virtual palpation of pelvic and femoral bony landmarks, (3) atlas-based morphing of muscle attachments, (4) automated selective cut of segmented muscle volumes and (5) sub-volume comparison. More details are provided in the following sections.

**Fig. 2:**
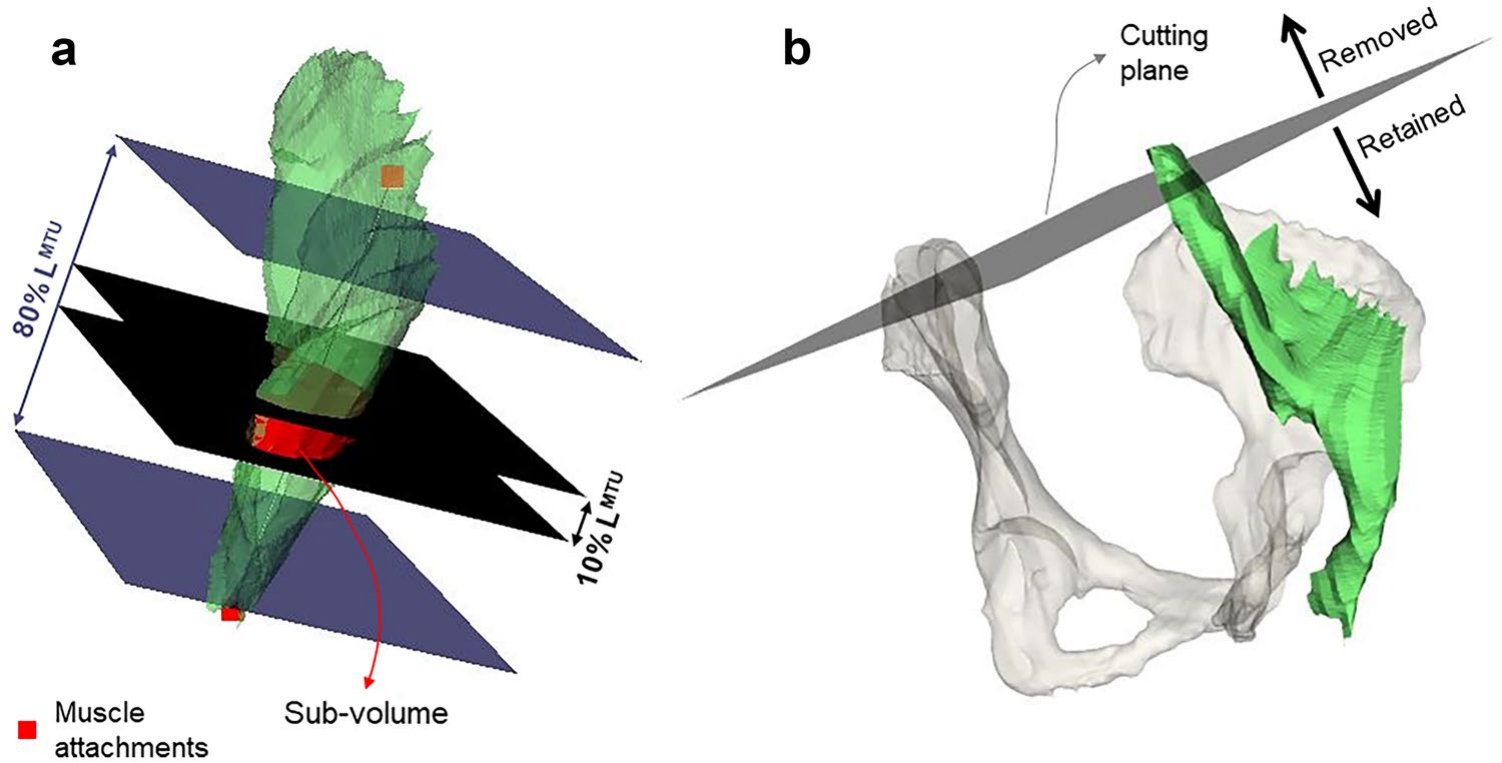
**a** Selective cut of the gluteus medius muscle volume (green). At each iteration, top and bottom cutting planes were iteratively moved up or down along the line connecting muscle origin and insertion points (red squares) in 5% steps of the muscle length (black = 10%, dark blue = 80%). **b** Iliopsoas muscle standardization performed prior to the definition of sub-volumes

For the iliopsoas muscle, an additional step (step 3b, Fig. 1) was performed to standardize the segmented volumes prior to proceed with the analyses. A cutting plane normal to the origin-to-insertion line and passing through the bony landmarks identified on the pelvic bone (i.e., on the left and right iliac crests) was defined and the proximal end of the muscle was removed (Fig. 2b). This step was deemed necessary to remove any possible bias due to artefacts on the images at the edges of the captured volume, which differed from patient to patient. These could affect contour identification in first place, and in turn all subsequent analyses and the repeatability measures.

### Manual Segmentation

The MRI data, stored in DICOM format, were imported in the Mimics Innovation Suite v22 (Materialise, Leuven, BE), and anonymized. The Multiple Slice Edit tool was then used to draw the contours of the left and right iliopsoas and gluteus medius muscles. All pixels enclosed in a contour were assigned to a 2D mask, specific to a structure of interest. This process, namely manual segmentation, was performed on each (coronal) slice. Automatic interpolation finally filled the gaps between consecutive segmentations, enabling the generation of 3D objects off the resulting 2D masks. One trained operator performed three repeated segmentations for all subjects and muscles of interest (gluteus medius: *n* = 40; iliopsoas: *n* = 34), on different and non-consecutive days, selecting the MRI data and target muscle to be segmented in a random fashion to minimize the memory effect. Similarly, to further enable a reproducibility assessment of the procedure, two additional operators (with different background and/or level of expertise compared to the first operator) (Table S2, Supplementary material) performed manual segmentations of the gluteus medius and iliopsoas muscles.

### Virtual Palpation

Using the free-software 3D Slicer [29], twelve points, corresponding to pre-selected anatomical bony landmarks on the pelvis and femurs, were manually identified on all MRIs via virtual palpation [30]. The 3D coordinates were then exported into text files for later use.

### Muscle Point Morphing

Since muscle aponeuroses and attachment areas were not clearly visible on MRIs, muscle origin and insertion points were mapped to the medical imaging data from a generic atlas (i.e., gait2392 OpenSim model [31–33]). Point morphing was performed in nmsBuilder [34], where the single value decomposition method was used to determine an affine transformation able to register corresponding pairs of bony landmarks (i.e., selected on both the gait2392 model and the MRIs, for each subject). The same transformation was then applied to all generic muscle points of interest (i.e., for the gluteus medius muscle: origin and insertion of the medial bundle; for the iliopsoas: origin of the psoas and insertion of the iliacus on the femur). Visual inspections were conducted to check for points (mis)placement. If deemed necessary (e.g., points not laying on the bone surfaces), the muscle attachments were snapped to the bone surface, i.e. (re)located to the nearest plausible surface point, through an automated procedure in MATLAB. A visual check was finally performed to ensure that the updated locations were in agreement with the underlying MRI.

### Sub-volume Definition

All segmented muscle volumes were divided in sub-volumes. Starting from the mid-point between muscle attachments, two parallel cutting planes, orthogonal to the line connecting origin and insertion points, were iteratively moved up and down, respectively, along the muscle line of action in 5% steps (of the muscle length) (Fig. 2a). At each iteration, only the volume included between the planes was preserved. Ten (sub)volumes per segmentation were thus generated. To ensure consistency, the process was fully automated via custom-written functions and scripts compiled in Python (v3.6).

### Data Analysis

For the repeatability assessment, for each subject and muscle, corresponding sub-volumes were compared using different metrics, in line with previous studies that assessed the reliability and/or repeatability of anatomical structures segmented on MRI images [35, 36]. First, the volume variance between repetitions was calculated. Values were normalized to the mean muscle (sub)volume and reported as percentage. Then, surface and shape similarity were quantified by computing the maximal Hausdorff distance (HD) and the Jaccard index (JI) [37], testing all possible combinations (i.e., repetitions: 1^st^ vs 2^nd^, 2^nd^ vs 3^rd^, 1^st^ vs 3^rd^). Mean HD and JI values (across the three combinations) were ultimately extracted. This enabled the quantification of the segmentation error and its distribution along the muscle volume. All operations were performed in Python using the stl-mesh and gias2 modules.

For the reproducibility assessment, the overall muscle volumes segmented by the three operators were extracted and compared.

### Statistical Analysis

Data were checked for normality. If data distributions were normal, a one-way repeated measures ANOVA was performed to compare Jaccard index, maximal Hausdorff distance and volume variance between cutting levels, i.e. depending on the amount of muscle volume accounted for. Post hoc analyses were conducted using paired *t-*tests implementing Bonferroni corrections to account for multiple comparisons. If data were not normally distributed, all metrics were compared using a Friedman test for repeated measures followed by a Wilcoxon signed-rank test. Statistical significance was initially set to *α* = 0.05. The inter-operator reproducibility was assessed by computing the intraclass correlation coefficient (i.e., ICC(1,1) and ICC(3,1)). All analyses were conducted in Python 3.6, using the Pingouin module [38]. Furthermore, linear mixed models (LMM) were employed to understand whether patients’ etiology affected (intra-operator) repeatability and (inter-operator) reproducibility. This last analysis was conducted in R (v4.2.1) using the rptR package (v0.9.22)[39], comparing the segmentations (of overall muscle volumes) performed by the three operators.

## Results

Overall, repeated muscle segmentations showed a moderate to high level of agreement. Nonetheless, for both muscles and all metrics, the statistical analyses (i.e., one-way repeated measures ANOVA or Friedman tests) revealed a significant main effect of the amount of volume accounted for (*P* < 0.013 for all tests). Post hoc analyses were thus performed, to identify sub-volume differences. All *P*-values reported in the following sections refer to the results of the post hoc analyses.

The reader is referred to the Supplementary Material for the results of the additional analyses (Table S2 for the reproducibility assessment, Table S3 for the effect of etiology on inter- and intra-operator assessments).

### Jaccard Index

**Fig. 3:**
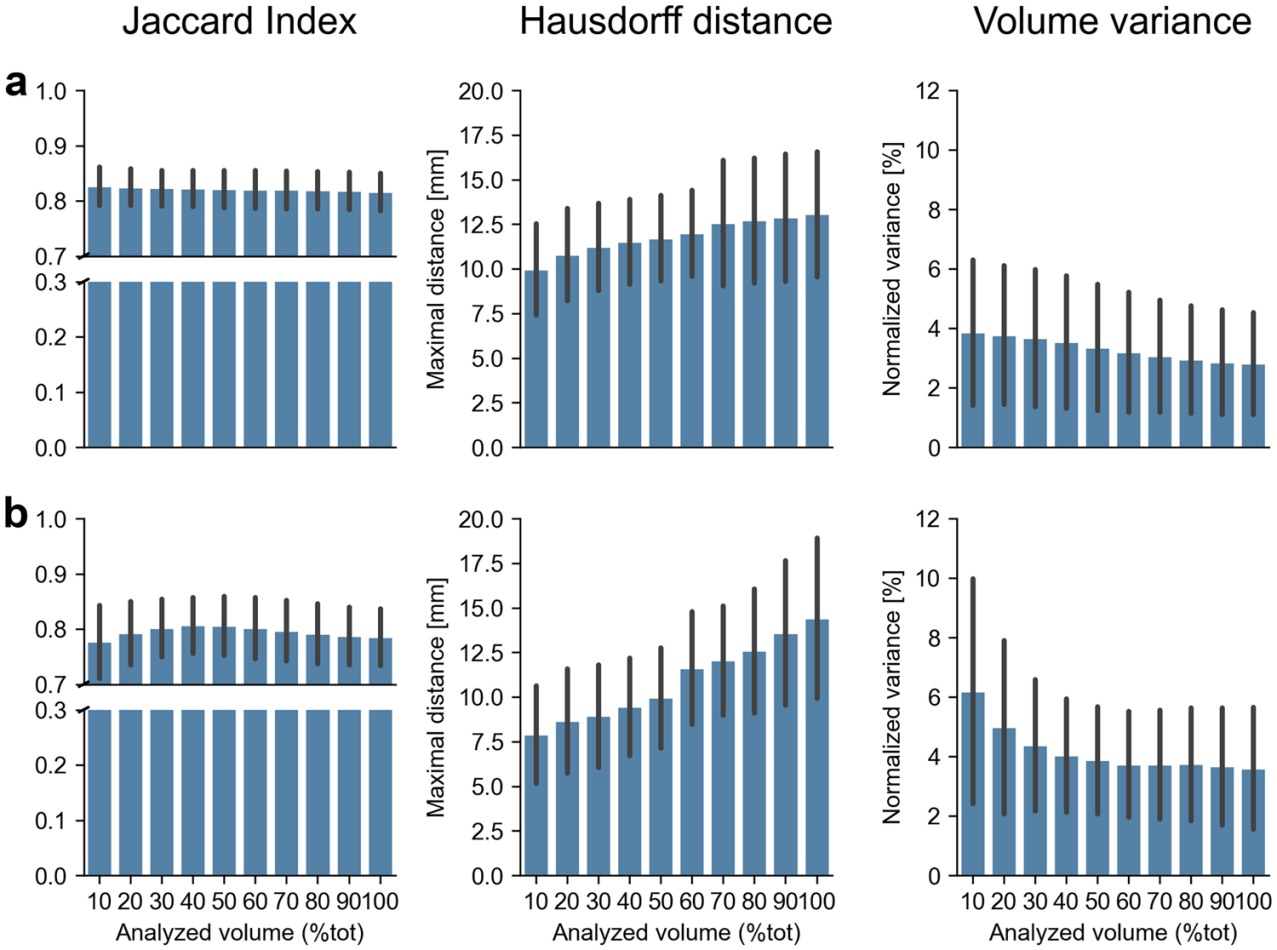
Similarity metrics (Jaccard index, Hausdorff distance and variance of the volume) selected to quantify the repeatability of manual segmentations of muscles on MRI. Results for the gluteus medius muscle (**a**) and for the iliopsoas muscle (**b**) are reported as mean (bar) and standard deviation (line). mm = millimeters

Shape-wise, all repeated segmentations, presented a good level of agreement. For the gluteus medius muscle, the Jaccard index was on average larger than 0.8 (i.e., 0.821 ± 0.03, min = 0.816, max = 0.827), slightly decreasing with the amount of volume analyzed (Fig. 3).

**Fig. 4:**
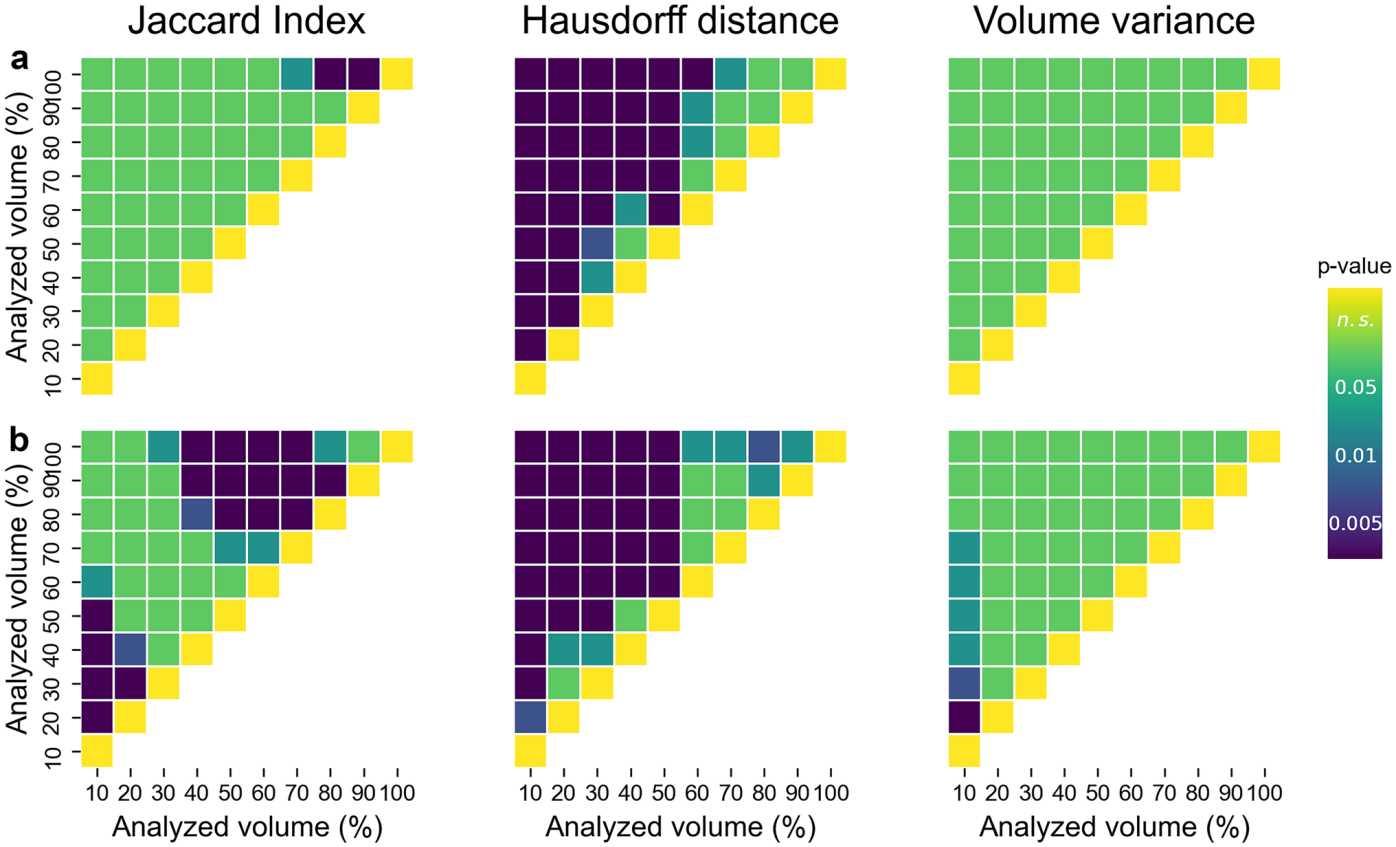
Results of the post hoc analysis (pairwise *T*-tests) performed to assess surface, shape and volume similarity between segmented muscle (sub)volumes (i.e., Jaccard index, Hausdorff distance and normalized volume variance), for (**a**) the gluteus medius and (**b**) the iliopsoas muscle. Each box within a subplot represents an individual comparison between cutting levels (e.g., between segmentations including 10% and 20% of the overall muscle volume). Green: *P* ≥ 0.05 (not significant), light blue: *P* < 0.05, blue: *P* ≤ 0.01, dark blue: *P* ≤ 0.005

Complete segmentations showed a significantly lower level of similarity compared to segmentations including 70%, 80% or 90% of the overall muscle volume (*P* = 0.045, *P* = 0.001, *P* = 0.000, respectively. Fig. 4a). 

For the iliopsoas muscle, the amount of volume included in the analysis had a more substantial effect on the Jaccard index, which was generally lower compared to the values observed for the gluteus medius (i.e., on average: 0.795 ± 0.09, min = 0.777, max = 0.807). Specifically, repeated segmentations were significantly more similar to one another (i.e., showed higher JI values) when only the middle portion of the muscle belly (i.e., central 40% to 70% of the muscle volume) was accounted for, compared to analyses including 80–100% of the overall volume (*P*_40_ < 0.009, *P*_50_ < 0.002, *P*_60_ = 0.001, *P*_70_ < 0.001. Fig. 4b, Table 2).

**Table 2:** Comparative metrics to assess the repeatability of manual segmentations

Analyzed volume (% muscle length)	Gluteus medius			Iliopsoas		
	JI	HD (mm)	nVV (%)	JI	HD (mm)	nVV (%)
10	0.827 (0.748,0.883)	9.996 (5.828,15.675)	3.864 (0.223,11.858)	0.777 (0.612,0.873)	7.903 (4.431,17.530)	6.198 (0.919,14.374)
20	0.825 (0.747,0.883)	10.620^†^ (6.266,18.559)	3.777 (0.538,11.878)	0.793^†v^ (0.645,0.868)	8.669^^^ (5.026,34.431)	4.995^†^ (0.553,11.121)
30	0.823 (0.746,0.884)	11.070^†^ (6.925,18.144)	3.670 (0.174,11.721)	0.802^†^ (0.668,0.883)	8.937 (5.337,175.270)	4.378 (0.563,8.880)
40	0.822 (0.747,0.884)	11.333^*^ (7.646,18.036)	3.542 (0.642,11.173)	0.807 (0.678,0.892)	9.461^*^ (5.829,34.431)	4.039 (0.402,7.706)
50	0.821 (0.749,0.885)	11.576 (7.599,33.859)	3.360 (0.379,10.280)	0.806 (0.664,0.892)	9.962 (5.941,37.300)	3.879 (0.707,8.004)
60	0.821 (0.745,0.887)	11.870^†^ (7.599,33.859)	3.196 (0.074,9.221)	0.802 (0.650,0.878)	11.629^†^ (7.022,35.316)	3.742 (0.263,7.579)
70	0.820 (0.743,0.888)	12.025 (7.599,34.707)	3.064 (0.518,8.404)	0.797^*^ (0.645,0.869)	12.057 (7.107,35.316)	3.741 (0.369,7.833)
80	0.820 (0.745,0.889)	12.189 (7.726,34.707)	2.954 (0.177,8.023)	0.792^†^ (0.645,0.867)	12.598 (7.810,35.316)	3.746 (0.644,8.587)
90	0.819 (0.739,0.889)	12.371 (7.726,34.707)	2.866 (0.244,7.785)	0.788^†^ (0.651,0.860)	13.597^*^ (7.864,35.316)	3.672 (0.580,9.054)
100	0.816^†^ (0.736,0.887)	12.592 (8.571,34.707)	2.814 (0.428,7.537)	0.786 (0.659,0.857)	14.423^*^ (8.206,35.316)	3.605 (0.687,9.457)

Results are reported as mean (min, max) values of the entire analyzed population (*n*_gluteus_ = 40, *n*_iliopsoas_ = 34)

Symbols indicate statistical significance, as detected by post hoc pairwise comparisons (with respect to the preceding row, e.g. 100 vs 90)

*JI* Jaccard Index, *HD* Hausdorff distance, *nVV* normalized volume variance, *mm* millimetres

* = *P* < 0.05, ^ = *P* ≤ 0.01, † = *P* ≤ 0.005

### Hausdorff Distance

The amount of selected volume further influenced the maximal Hausdorff distance: the larger the analyzed volume, the larger the discrepancy between repeated segmentations. This effect was more noticeable for the iliopsoas (HD = 10.92 ± 2.13 mm, min = 7.90 mm, max = 14.42 mm. Fig. 3, Table 2) than for the gluteus medius muscle (HD = 11.56 ± 0.78 mm, min = 10.00 mm, max = 12.59 mm. Fig. 3, Table 2). In general, the surface-to-surface distance error between repeated segmentations was the lowest (*P*_ilps_ < 0.01, *P*_gmed_ < 0.002) when only a minimal amount of segmented muscle volume (i.e., within ± 5% of the muscle–tendon unit length from the centre of the muscle) was analyzed. For the gluteus medius, the post hoc analysis revealed no significant differences between analyses including over 80% of the entirely segmented muscle volume (Fig. 4a). Nonetheless, these were associated to the largest HD values overall. For the iliopsoas, the inclusion of muscle extremities (i.e., full segmentations) led to the largest measured surface-to-surface errors (*P*_100_ ≤ 0.025, compared to all other analyses. Fig. 4b).

### Volume Variance

In terms of volume, all repeated segmentations were comparable to one another, independently on the amount of volume accounted for. On average, the variance of the volume, which was normalized to the corresponding mean muscle volume to allow for comparisons, was lower than 4% and 6.5% for the gluteus medius and the iliopsoas muscle, respectively. More specifically, for the gluteus medius, the cutting level did not show any noticeable effect on the results, as revealed by the statistical analysis (*P* > 0.05, for all comparisons. Fig. 4a). On the other hand, for the iliopsoas muscle, there was one exception: repeated segmentations of muscle volumes corresponding to the central 10% of the muscle belly showed significantly larger variance compared to segmentations including up to 70% of the overall muscle volume (*P* < 0.03, Fig. 4b).

## Discussion

The aims of this study were (1) to assess the repeatability of manual segmentations of the gluteus medius and iliopsoas muscles on standard 1.5 T MRIs and (2) to determine whether the segmentation error was equally distributed across the volume or confined in specific areas (e.g., muscle extremities). To this end, forty gluteus medius and thirty-four iliopsoas muscles were manually segmented by one trained operator using the Mimics software (v.22). All segmentations were performed three times in non-consecutive days and compared using three different metrics: JI as measure of shape similarity, maximal HD to quantify surface-to-surface error, and normalized volume variance (nVV) to determine volumetric differences. To identify the areas more prone to segmentation error, the analysis was repeated on portions of the segmented muscle volumes (i.e., ten sub-volumes of incremental size), which were automatically generated in Python.

In agreement with our first hypothesis (H1), repeated manual segmentations of the gluteus medius and iliopsoas muscles showed a high level of similarity (i.e., JI ~ 0.8, HD < 15 mm and normalized volume variance 2–6%). Noticeably, the Jaccard indices ranged between 0.777 and 0.807 for the iliopsoas, and between 0.816 and 0.827 for the gluteus medius muscle. These results further demonstrate that the manual segmentation of soft tissues on MRIs is not only possible, but also repeatable. The identification of muscle parameters from manually segmented muscle volumes, which is of outmost importance for musculoskeletal modelling and clinical applications, can be considered affected by minimal (if not negligible) uncertainty due to the segmentation procedure.

Nonetheless, distinctions need to be drawn. In fact, while HD and nVV did vary similarly for both muscles (i.e., increasing and decreasing, respectively, the larger the portion of the analyzed volume was), this did not hold true for the JI metric. For the gluteus medius, JI slightly reduced the more volume was accounted for, and the differences were statistically significant only between full segmentations and segmentations including over 70% of the muscle volume. On the other hand, for the iliopsoas muscle, considering little (< 30%) or large (> 70%) portions of muscle volume resulted in significantly lower volume similarity compared to analyses including 40–70% of the overall muscle volume. This is likely due to the simpler anatomical structure characterizing the gluteus medius muscle compared to the iliopsoas, as hypothesized (H2).

Last, as hypothesized (H3), the observed level of similarity was highest when muscle extremities were not included in the analysis. Full segmentations showed lowest JI and largest surface-to-surface distance errors. Interestingly, for the iliopsoas, volume variability (i.e., normalized variance) was largest when only the central portion (i.e., 10%) of the segmentation was considered. This is likely due to the shape of the iliopsoas muscle that in its central portion attaches to and wraps around the iliac crests, adding complexity to the process of contour identification. Extreme care should be taken when segmenting complex structures, as segmentation inaccuracies may be further enhanced while interpolating consecutive 2D segmentations to generate 3D (volume) reconstructions.

### Limitations

This study has few limitations. First, what in the “Results” section we referred to as full segmentations for the iliopsoas muscle, were in fact standardized muscle volumes. Therefore, for the iliopsoas muscle only, the analysis may have not fully captured all discrepancies between repeated segmentations, as the proximal end of the muscle (typically more difficult to identify on MRI) was not included. Nonetheless, the standardization was required as the MRI data used in this study were retrospectively collected from the institutional database, therefore the images were not homogeneous in terms of scanned volume, possibly affecting comparisons. Second, while most of the acquisitions shared the same spatial resolution and slice thickness, in some cases, the above parameters slightly differed, potentially increasing or reducing the precision of manual segmentations. Third, the dataset included both healthy and affected muscles, as patients’ data were segmented bilaterally. Due to an altered composition, diseased muscle tissues may appear less clearly on MRIs compared to healthy muscles, negatively affecting repeatability metrics. Furthermore, the analyzed data belonged to patients with different etiology, resulting in a small sample size per diagnosis group. However, statistical analyses using linear mixed models showed that patient’s etiology had a limited effect on the repeatability and reproducibility of manual muscle segmentations, for both the iliopsoas and gluteus medius muscles. Finally, it must be noted that all data were acquired prior to 2015, for diagnostic and clinical purposes (i.e., not optimised for research). This may have affected image quality, possibly limiting the operator’s ability to precisely identify muscle contours. Therefore, the hereby reported level of accuracy may be slightly underestimating what can be currently achieved on higher quality imaging data.

## Conclusions

This study aimed to assess the repeatability of manual segmentations of the iliopsoas and gluteus medius muscles on diagnostic 1.5 T MRIs. To this end, one operator performed repeated manual segmentations of the muscles of interest on axial T1-weighted MRI scans of the pelvic area (hip), retrospectively collected from the database of the institute (*n*_ilps_ = 34, *n*_gmed_ = 40). Our results show that 3D muscle volumes reconstructed from the interpolation of consecutive manual 2D segmentations are highly repeatable, in terms of shape similarity (JI > 0.77), surface similarity (maximal HD < 15 mm) and volume variance (nVV < 6.5%). Hence, the slice-by-slice manual segmentation of muscles on MRIs should be considered both for musculoskeletal modelling applications (to extract parameters of interest towards model personalization), and clinical applications (e.g., in the assessment of sarcopenia). Nonetheless, extreme care should be taken when segmenting complex structures or muscles wrapping around bones, as contour identification becomes non-trivial and susceptible to errors that could be magnified during interpolation, reducing the overall accuracy.

## Supplementary Information

Below is the link to the electronic supplementary material.

Supplementary file1 (DOCX 31 KB)

## Declarations

### Disclaimer

Content in this publication reflects the authors’ view and neither IMI nor the European Union, EFPIA, Italian Ministry of Health or any Associated Partners are responsible for any use that may be made of the information contained herein.
