# Supplementary material for: Intra-operator Repeatability of Manual Segmentations of the Hip Muscles on Clinical Magnetic Resonance Images
Source: J Digit Imaging. 2022 Oct 11;36(1):143–52. doi: 10.1007/s10278-022-00700-0 (PMC9984589; doi:10.1007/s10278-022-00700-0)
Supplement: Supplementary file 1 — Supplementary file1 (DOCX 31 KB) [file 10278_2022_700_MOESM1_ESM.docx]

# SUPPLEMENTARY MATERIAL

**Table S1** Acquisition sequence parameters and patient demographics

| Subject  ID | Image information | | | | | | Patient | | |
| --- | --- | --- | --- | --- | --- | --- | --- | --- | --- |
|  | TE  (ms) | TR (ms) | Inversion Time (ms) | Train Length | Sequence | FOV (mm) | Mass  (kg) | BMI  (kg·m^2^) | Sex |
| S01 | 11.38 | 500 | 0 | 3 | Cor T1 FSE | 370.02 | 47 | 24.5 | F |
| S02 | 10.72 | 420 | 0 | 3 | Cor T1 FSE | 380.01 | 70 | 16.9 | F |
| S03 | 10.73 | 500 | 0 | 3 | Cor T1 FSE | 419.99 | 85 | 25.0 | M |
| S04 | 10.79 | 460 | 0 | 3 | Cor T1 FSE | 419.99 | 71 | 25.3 | F |
| S05 | 10.54 | 580 | 0 | 3 | Cor T1 FSE | 459.98 | 75 | 24.8 | F |
| S06 | 11.24 | 520 | 0 | 3 | Cor T1 FSE | 419.99 | 76 | 26.5 | M |
| S07 | 10.59 | 500 | 0 | 3 | Cor T1 FSE | 440.01 | 68 | 37.3 | F |
| S08 | 10.49 | 500 | 0 | 3 | Cor T1 FSE | 380.01 | 70 | 25.6 | F |
| S09 | 10.41 | 620 | 0 | 4 | Cor T1 FSE | 480 | 90 | 29.4 | F |
| S10 | 11.24 | 520 | 0 | 3 | Cor T1 FSE | 419.99 | 50 | 23.3 | F |
| S11 | 9.90 | 480 | 0 | 3 | Cor T1 FSE | 400.03 | 49 | 25.8 | F |
| S12 | 10.66 | 500 | 0 | 3 | Cor T1 FSE | 419.99 | 70 | 26.5 | F |
| S13 | 10.36 | 620 | 0 | 4 | Cor T1 FSE | 419.99 | 98 | 21.2 | M |
| S14 | 18.00 | 1151.54 | - | 3 | Cor T1 TSE | 420 | 76 | 21.0 | M |
| S15 | 10.86 | 600 | 0 | 3 | Cor T1 FSE | 419.99 | 68 | 17.3 | F |
| S16 | 10.74 | 480 | 0 | 4 | Cor T1 FSE | 380.01 | 73 | 18.8 | M |
| S17 | 11.25 | 520 | 0 | 3 | Cor T1 FSE | 440.01 | 83 | 27.7 | M |
| S18 | 10.49 | 500 | 0 | 3 | Cor T1 FSE | 419.99 | 63 | 24.5 | F |
| S19 | 10.75 | 520 | 0 | 3 | Cor T1 FSE | 419.99 | 70 | 29.1 | M |
| S20 | 10.56 | 580 | 0 | 3 | Cor T1 FSE | 440.01 | 75 | 29.2 | M |

BMI = body mass index, F = female, M = male, FOV = field of view, FSE = fast spin echo, TE = echo time, TR = repetition time, TSE = turbo spin echo

**Table S2** Inter-operator reproducibility for the gluteus medius and iliopsoas muscles, assessed on 20 muscles (n=10 subjects, randomly selected) segmented by three different operators with different background and level of expertise with manual segmentations. Segmentations were compared in terms of overall muscle volume.

| Operators details | | | | | | | |
| --- | --- | --- | --- | --- | --- | --- | --- |
| Operator id | | Background | | | Level | | |
| 1 | | Medicine (Orthopaedics) | | | Expert | | |
| 2 | | Biomedical Engineering | | | Expert | | |
| 3 | | Exercise Sciences | | | Non-expert | | |
| Gluteus Medius | | | | | | | |
| Type | Description | | ICC | F | | pval | CI95% |
| ICC (1,1) | Single raters absolute | | 0.757 | 10.359 | | <0.0001 | [0.57-0.89] |
| ICC (2,1) | Single random raters | | 0.769 | 27.063 | | <0.0001 | [0.31-0.92] |
| ICC (3,1) | Single fixed raters | | 0.897 | 27.063 | | <0.0001 | [0.80-0.95] |
| ICC (1,2) | Average raters absolute | | 0.903 | 10.359 | | <0.0001 | [0.80-0.96] |
| ICC (2,2) | Average random raters | | 0.909 | 27.063 | | <0.0001 | [0.57-0.97] |
| ICC (3,2) | Average fixed raters | | 0.963 | 27.063 | | <0.0001 | [0.92-0.98] |
| Iliopsoas | | | | | | | |
| Type | Description | | ICC | F | | pval | CI95% |
| ICC (1,1) | Single raters absolute | | 0.693 | 7.757 | | <0.0001 | [0.48-0.85] |
| ICC (2,1) | Single random raters | | 0.716 | 38.970 | | <0.0001 | [0.12-0.91] |
| ICC (3,1) | Single fixed raters | | 0.927 | 38.970 | | <0.0001 | [0.85-0.97] |
| ICC (1,2) | Average raters absolute | | 0.871 | 7.757 | | <0.0001 | [0.73-0.94] |
| ICC (2,2) | Average random raters | | 0.883 | 38.970 | | <0.0001 | [0.30-0.97] |
| ICC (3,2) | Average fixed raters | | 0.974 | 38.970 | | <0.0001 | [0.95-0.99] |
| CI = confidence interval, ICC = intraclass correlation coefficient, pval = p-value | | | | | | | |

**Table S3** Effect of etiology on intra and inter-operator repeatability measures. The analyses were conducted in the statistical software R, using the rptR package (v0.9.22) to generate linear mixed-models. Repeated (full) muscle segmentations performed by two experienced operators were compared. For all subjects, only the muscles on the affected side(s) were included in the analysis (n_gmed_ = 22, n_ilps_ = 19). Two patients were bilaterally affected.

| Linear Mixed Model | | | | | |
| --- | --- | --- | --- | --- | --- |
| Volume ~ Etiology + (1\|sID) | | | | | |
| Repeatability  (1 Experienced Operator – Medical background, 3 repeated segmentations) | | | | | |
| Muscle | Term | R | SE | CI95% | P |
| Gluteus medius | sID | 0.832 | 0.120 | [0.486-0.931] | <0.0001 |
|  | Etiology | 0.117 | 0.123 | [0.025-0.465] | - |
| Iliopsoas | sID | 0.987 | 0.098 | [0.613-0.977] | <0.0001 |
|  | Etiology | 0.001 | 0.098 | [0.013-0.366] | - |
| Repeatability  (1 Experienced Operator – Engineering background, 2 repeated segmentations) | | | | | |
| Muscle | Term | R | SE | CI95% | P |
| Gluteus medius | sID | 0.83 | 0.111 | [0.504-0.920] | <0.0001 |
|  | Etiology | 0.08 | 0.108 | [0.015-0.430] | - |
| Iliopsoas | sID | 0.80 | 0.131 | [0.433-0.932] | <0.0001 |
|  | Etiology | 0.135 | 0.129 | [0.030-0.515] | - |
| Reproducibility  (2 Experienced Operators, 1 segmentation) | | | | | |
| Muscle | Term | R | SE | CI95% | P |
| Gluteus medius | sID | 0.57 | 0.138 | [0.247-0.776] | 0.00216 |
|  | Etiology | 0.083 | 0.101 | [0.018-0.385] | - |
| Iliopsoas | sID | 0.578 | 0.146 | [0.225-0.795] | 0.00185 |
|  | Etiology | 0.137 | 0.122 | [0.019-0.470] | - |
| CI = confidence interval, gmed = gluteus medius, ilps = iliopsoas, P = p-value, R = repeatability, SE = standard error, sID = subject ID | | | | | |
